# Dysregulation of the TLR4/MyD88/NF‐κB Signaling Pathway and Pro‐Inflammatory Cytokines in First‐Episode Major Depressive Disorder: A Cross‐Sectional Case–Control Study

**DOI:** 10.1002/iid3.70477

**Published:** 2026-06-25

**Authors:** Yaser Mohammadi, Omid Kooshkaki, Aliakbar Esmaeili, Hasti Anani Sarab, Gholamreza Anani Sarab

**Affiliations:** ^1^ Student Research Committee Iran University of Medical Sciences Tehran Iran; ^2^ Department of Biochemistry, School of Medicine Iran University of Medical Sciences Tehran Iran; ^3^ Department of Medical Immunology, Faculty of Medicine Birjand University of Medical Sciences Birjand Iran; ^4^ Psychiatry and Behavioral Science Research Center Birjand University of Medical Sciences Birjand Iran; ^5^ Faculty of Medicine Üsküdar University Istanbul Turkey

**Keywords:** cytokines, inflammation, inflammatory biomarkers, major depressive disorder (MDD), psychoneuroimmunology

## Abstract

**Introduction:**

Inflammation is increasingly recognized as a key contributor to the development and progression of major depressive disorder (MDD). This case–control study aims to assess the expression of inflammatory pathway components (TRIF, MyD88, NF‐κB) and serum levels of TNF‐α and IL‐6 in remitted and untreated MDD patients compared to healthy controls.

**Methods:**

This study included 150 participants: 50 newly diagnosed and untreated MDD patients, 50 MDD patients who achieved complete clinical remission following at least 8 weeks of continuous TCA treatment (TCA remitters), and 50 matched healthy controls. MDD diagnosis was based on DSM‐V criteria and Beck Depression Inventory (BDI). Peripheral blood samples were collected to isolate serum and PBMCs. mRNA expression of NF‐κB, TRIF, and MyD88 was quantified by qRT‐PCR, while serum levels of TNF‐α and IL‐6 were measured using ELISA. Data were analyzed using one‐way ANOVA with Tukey's post hoc test, considering *p* < 0.05 as statistically significant.

**Results:**

No significant differences were found in age or gender among the three groups, confirming demographic comparability. BDI scores were markedly higher in both untreated and remitted MDD patients compared to controls, with untreated patients showing the most severe symptoms. Gene expression analysis revealed a significant upregulation of MYD88 (~5‐fold) and NF‐κB (~2.5‐fold) in untreated MDD patients compared to controls (*p* < 0.001); treated patients also showed higher expression, though to a lesser extent. TRIF expression was higher in treated patients, but not significantly. Serum levels of TNF‐α and IL‐6 were significantly higher in untreated MDD patients versus controls (*p* < 0.001); IL‐6 and TNF‐α levels were also higher in treated patients but significantly lower than in the untreated group.

**Conclusion:**

The results support the hypothesis of a link between immune dysregulation and MDD. However, further human studies are necessary to validate the findings of the present study.

## 1 Introduction

Major depressive disorder (MDD) is among the most prevalent and debilitating psychiatric disorders globally. According to the World Health Organization, MDD is projected to become the second leading cause of global disease burden after ischemic heart disease by the year 2030 [1, 2]. Women are more frequently diagnosed with MDD than men [3]. Despite extensive research, the exact pathophysiology of MDD remains elusive. A growing body of evidence, however, suggests that psychological stress may trigger immune responses through activation of inflammatory pathways and modulation of immune cell trafficking via stress‐induced central nervous system (CNS) signals [4, 5].

While inflammation has been well established in the pathogenesis of several chronic diseases, such as rheumatoid arthritis, atherosclerosis, obesity, diabetes, and cardiovascular conditions [6], its precise role in MDD is still being explored. It is believed that stress‐induced inflammation, though initially adaptive, can lead to prolonged immune activation, which in turn may alter neurochemical signaling and contribute to the development of depressive symptoms. These symptoms often include low mood, anhedonia, cognitive impairments, fatigue, reduced energy, and social withdrawal [7].

For instance, Lindqvist et al. reported higher interleukin‐6 (IL‐6) levels in the cerebrospinal fluid (CSF) of individuals who attempted suicide compared to both depressed non‐attempters and healthy controls [8]. Similarly, Shelton et al. demonstrated upregulation of multiple pro‐inflammatory cytokines in postmortem brain tissues of MDD patients [9]. These findings point toward a potential link between peripheral inflammation, immune system activation, and psychiatric disorders, particularly depression [10].

Toll‐like receptors (TLRs) play a pivotal role in initiating and modulating inflammatory responses [11]. As key members of the pattern recognition receptors family, TLRs recognize pathogen‐associated molecular patterns and damage‐associated molecular patterns, thereby mediating innate and adaptive immune responses [12]. Among them, Toll‐like receptor 4 (TLR4) is well‐characterized for its ability to sense lipopolysaccharides (LPS) and initiate inflammatory signaling cascades [13]. Importantly, TLR4 has been implicated in CNS inflammation and in the regulation of depression‐ and anxiety‐like behaviors in murine models [14].

Downstream of TLR4 activation, adaptor proteins such as myeloid differentiation primary response 88 (MyD88) and TIR‐domain‐containing adapter‐inducing interferon‐β (TRIF) propagate intracellular signaling events. These cascades involve activation of key transcription factors like NF‐κB through intermediary molecules such as IRAK1 and TRAF6, ultimately resulting in higher production of pro‐inflammatory cytokines and chemokines, including TNF‐α, IL‐1β, IL‐6, CCL2, and CXCL6 [15, 16].

While C‐reactive protein (CRP) is widely recognized as a sensitive systemic marker of inflammation and has been extensively studied in depression research [17, 18], the current investigation focuses specifically on upstream regulatory mechanisms of the inflammatory cascade. Our rationale for examining the TLR4/MyD88/NF‐κB pathway components stems from evidence suggesting that these signaling molecules may represent primary initiators of inflammatory responses in MDD, rather than downstream consequences [19, 20]. Furthermore, previous studies have shown that TLR4‐mediated signaling can be activated independently of peripheral inflammatory markers like CRP, particularly in neuroinflammatory conditions [21, 22]. Given that first‐episode, treatment‐naïve MDD patients may exhibit distinct inflammatory profiles compared to chronic or treated cases, investigating proximal signaling elements may provide mechanistic insights into early disease pathogenesis [23, 24].

Given the critical involvement of the TLR4/MyD88/NF‐κB signaling pathway in modulating inflammation, it is hypothesized that dysregulation of this axis may contribute to the pathophysiology of MDD. Accordingly, the current study was designed to evaluate mRNA expression levels of TRIF, MyD88, and NF‐κB, as well as serum concentrations of TNF‐α and IL‐6, in both treated and untreated first‐episode MDD patients compared to healthy controls.

## 2 Material and Method

### 2.1 Patients

A total of 150 participants were enrolled in this study. Participants were recruited from the Psychology Clinic of Imam Reza Hospital, Birjand, Iran, which is the only specialized referral center for psychiatric disorders in South Khorasan Province. Fifty newly diagnosed MDD patients who had presented to the clinic for the first time, had not received any prior psychiatric or psychotherapeutic treatment, and were diagnosed by a board‐certified psychiatrist according to DSM‐V criteria were assigned to the “untreated” group. These patients were sampled prior to the initiation of any pharmacological treatment.

In parallel, 50 MDD patients who had already initiated antidepressant treatment as part of routine clinical care and had completed at least 8 weeks of continuous, documented therapy with tricyclic antidepressants (TCAs), including amitriptyline (75–150 mg/day), and who demonstrated complete clinical remission following treatment were assigned to the “TCA remitter” group. These patients were identified during regular follow‐up visits. Because only patients meeting remission criteria after TCA therapy were eligible for inclusion in this subgroup, recruitment required an extended enrollment period to identify an adequate number of responders under real‐world clinical conditions.

Additionally, 50 healthy individuals were selected as the control group and matched to the patient groups in terms of age, sex, ethnicity, and demographic characteristics. All MDD patients were evaluated using the Beck Depression Inventory (BDI) at the time of enrollment.

Importantly, treatment allocation and clinical management were determined entirely by treating psychiatrists as part of routine patient care and were independent of study participation. No patient was denied, delayed, or modified standard treatment for research purposes.

Patients with a history of autoimmune diseases, liver disorders, immunodeficiency conditions, acute or chronic infections, use of immunosuppressive drugs within the last 6 months, substance abuse, drug dependence, self‐medication, and individuals with any history of suicidal attempts or active suicidal ideation were excluded from the study. The Ethics Committee of Birjand University of Medical Sciences approved all study procedures, and all participants provided written informed consent prior to sample collection.

### 2.2 Serum and peripheral blood mononuclear cells (PBMCs) Isolation

After 8–10 h of fasting, peripheral blood samples of study groups were collected in 5.5 mL tubes pre‐coated with EDTA as anti‐coagulantand was immediately prepared for RNA extraction. Also, coagulated blood samples of study groups were collected, serums were taken, centrifuged at 2500 rpm for 10 min and were stored at −80°C until testing.

### 2.3 RNA Extraction and cDNA Synthesis

Total RNA was isolated from PBMCs using the total RNA extraction kit (Parstous biotechnology, Mashhad, Iran) according to the manufacturer's instructions. DNase was used to remove possible genomic DNA contaminations before cDNA synthesis (Jenabioscience, GmbH). The quality of RNA was verified by horizontal agarosegel electrophoresis. RNA quantity was evaluated by a Nanodrop 2000 spectrophotometer (BioTek, EPOCH). Total RNA was reverse transcripted into cDNA using Easy™ cDNA Synthesis Kit (Parstous biotechnology). The following programfor reverse transcription was performed using Setinan Eppendorf Mastercycler system (Germany Eppendorf 5345):65°C for 10 min ((without reverse transcription enzymes), −20°C for 1 min (cooling), 57°C for 60 min (added reverse transcription enzymes), and 95°C for 10 min (reverse transcription enzyme inactivation).

### 2.4 Quantitative Real‐Time PCR

Quantitative real‐time PCR was performed using specific primers for NF‐κB, TRIF, and MYD88 and run on the ABI Step One™ Real‐Time PCR System (Applied Biosystems; Thermo Fisher Scientific) and using the SYBR green master mix (Parstous biotechnology). β‐actin was used as an endogenous control. Primer sequences are shown in Table 1. The amplification was set as follows: 95°C for 10 min (1 cycle),95°C for 30 s, 60°C for 30 s, and 72°C for 30 s (40 cycles). All reactions were duplicated. Reaction mixtures, without RNA, were used as negative controls in each run. Fold changes expression were calculated using 2^−∆∆Ct^ method. Table 1 shows the sequences of the primers used.

**TABLE 1:** Oligonucleotides used in this study.

Gene	Forward primer	Reverse primer
NF‐kB	5'‐ CTGAACCAGGGCATACCTGT‐3'	5'‐ GAGAAGTCCATGTCCGCAAT ‐3'
TRIF	5'‐ ATCCCTGATCTGCTTGGGCA ‐3'	5'‐CGAAGGCGCTAGGAAGTGAT‐3'
MYD88	5'‐ TGGCACCTGTGTCTGGTCTA‐3'	5'‐ACATTCCTTGCTCTGCAGGT‐3'
B‐ACTIN	5'‐ TGGCACCCAGCACAATGAA‐3'	5'‐TAAGTCATAGTCCGCCTAGAAGCA‐3'

### 2.5 Cytokine Detection

Serum levels of IL‐6 and TNF‐alpha were measured by enzyme‐linked immunosorbent assay (ELISA) according to the manufacturer's instructions (Karmania Pars Gene) and analyzed at 450 nm wavelengths using the ELx808 microplate reader (Bio‐TeK). The minimum detection limit for IL‐6 and TNF‐alpha in our study was 3.5 and 4.6 pg/mL, respectively.

### 2.6 Statistical Analysis

All statistical analyses were performed using SPSS software (version 21.0, IBM). Data are presented as mean ± standard deviation (SD). Differences among the three study groups were assessed using one‐way analysis of variance (ANOVA), followed by Tukey's post hoc test for multiple comparisons. The *p*‐value of less than 0.05 was considered to be significant. Assumptions of normality and homogeneity of variance were checked prior to performing ANOVA.

## 3 Results

### 3.1 Demographic Information of the Participants

The demographic and clinical data of the three study groups—including the control group (*n* = 50), untreated MDD patients (*n* = 50), and TCA remitter group (*n* = 50)—are presented in Table 2. No statistically significant differences were observed among the groups in terms of age and gender, indicating demographic homogeneity across the study population.

**TABLE 2:** Demographic data for control subjects and major depressive patients (MDD).

Variable	Healthy control group (*n* = 50)	Untreated first‐episode MDD group (*n* = 50)	TCA remitter group (*n* = 50)	*p* value
Age (years)	38.37 ± 10.22	39.15 ± 13.52	43.38 ± 14.91	0.48
Sex (female/male)	35/15	35/15	35/15	0.178
BDI (mean ± SD)	9.56 ± 8.87	36.42 ± 10.90	24.47 ± 15.64	*< 0.05

*Note:* *p < 0.05 (untreated group vs. treated and control group).Data are presented as Mean ± SD. *N* = 50.

Abbreviations: BDI, Beck Depression Inventory (point score); MDD, major depressive disorder; TCA, tricyclic antidepressants.

Assessment of depression severity using the BDI revealed statistically significant differences among the groups (*p* < 0.05). The mean BDI score in the control group (9.56 ± 8.87) was significantly lower than that of the untreated group (36.42 ± 10.90) and the TCA remitter group (24.47 ± 15.64). Specifically, the BDI score in the untreated group was 280.96% higher than that of the control group, while the TCA remitter group showed a 155.96% higher BDI score compared to the control group. Furthermore, the TCA remitter group exhibited significantly lower BDI scores than untreated patients, with the BDI score in the untreated group being 48.84% higher than that of the TCA remitter group.

### 3.2 Effect on Inflammatory Gene Expression

**FIGURE 1:**
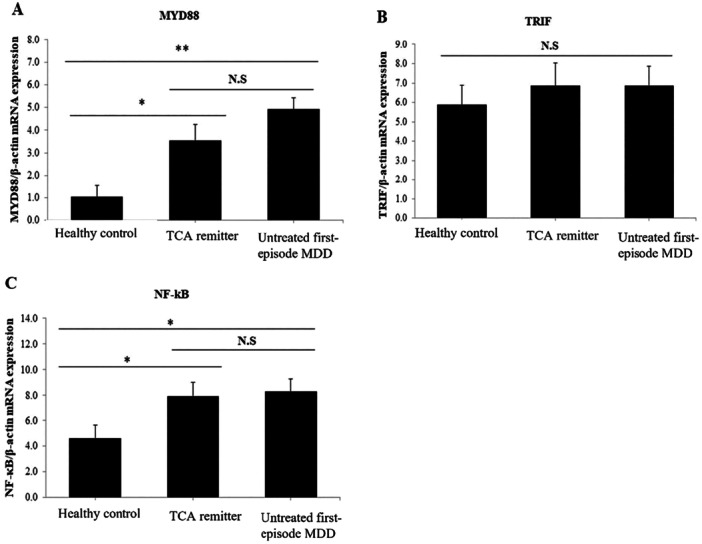
The mRNA expression levels of Myeloid Differentiation Primary Response 88 (MYD88), TRIF (TIR‐domain‐containing adapter‐inducing interferon‐β), and Nuclear Factor κappa‐B (NF‐κB). (A) MYD88, (B) TRIF, and (C) NF‐κB. Data are presented in mean ± SD of three separate groups. TCA, tricyclic antidepressants. *N* = 50. *:*p* < 0.05, **:*p* < 0.001.

The expression levels of MYD88, TRIF, and NF‐κB genes are illustrated in Figure 1.

Compared to the control group, MYD88 gene expression (Figure 1A) was significantly higher—by approximately fivefold—in the untreated MDD patient group (*p* < 0.001). In the TCA remitter group, MYD88 expression was also significantly higher—by approximately threefold—compared to the control group (*p* < 0.05). However, although MYD88 expression was higher in the untreated group compared to the TCA remitter group, this difference was not statistically significant.

TRIF gene expression (Figure 1B) was higher in the TCA remitter group compared to the other groups, although the difference was not statistically significant.

NF‐κB gene expression (Figure 1C) showed a similar pattern to MYD88. In the untreated MDD group, NF‐κB expression was significantly higher—approximately 2.5‐fold—compared to the control group (*p* < 0.001). In the TCA remitter group, NF‐κB expression was also significantly higher—about twofold—compared to the control group (*p* < 0.05). However, the difference between the TCA remitter and untreated groups was not statistically significant.

### 3.3 Effect on Serum Levels of Inflammatory Cytokines

**FIGURE 2:**
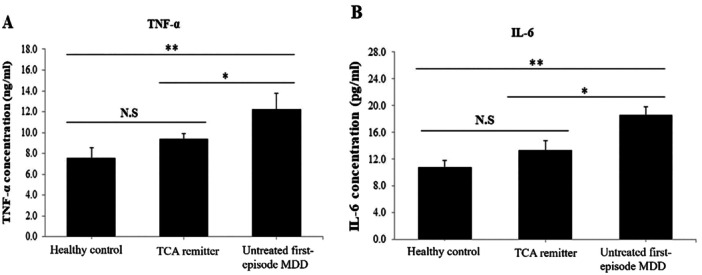
Serum levels of Tumor Necrosis Factor‐α (TNF‐α) and Interleukine‐6 (IL‐6) in the study groups. The serum levels of (A) TNF‐α and (B) IL‐6 were evaluated using Enzyme‐Linked Immunosorbent Assay (ELISA). TCA, Tricyclic antidepressants. Data are presented as Mean ± SD. *N* = 50. *p < 0.05, **p < 0.001.

The serum levels of the pro‐inflammatory cytokines TNF‐α and IL‐6 in the study groups are presented in Figure 2A,B, respectively.

Compared to the control group, serum TNF‐α levels were significantly higher in the untreated MDD patient group (*p* < 0.001). In the TCA remitter group, TNF‐α levels were also higher compared to controls, but the difference was not statistically significant. Notably, a statistically significant increase in TNF‐α levels was observed in the untreated group compared to the TCA remitter group (*p* < 0.05).

Serum IL‐6 levels were higher in both treated and untreated groups compared to the control group; however, this increase reached statistical significance only in the untreated group (*p* < 0.001). Moreover, IL‐6 levels were significantly higher in the untreated group than in the TCA remitter group (*p* < 0.05).

## 4 Discussion

The results of this study provide a comprehensive picture of the inflammatory status in patients with MDD and offer strong evidence for a significant association between inflammatory processes and this psychiatric condition. Demographic findings indicate that the three study groups were homogeneous in terms of age and gender, which strengthens the validity of between‐group comparisons. The significant differences in BDI scores between the groups—with a mean score of 9.56 in the control group, 36.42 in the untreated group, and 24.47 in the TCA remitter group—reflect the varying severity of depressive symptoms observed across clinically distinct groups, with lower scores in the TCA remitter group being consistent with their remitted clinical status rather than implying within‐subject change. Moreover, the results showed that the expression levels of inflammatory genes (MYD88, TRIF, and NF‐κB) and serum levels of inflammatory cytokines (TNF‐α and IL‐6) were significantly higher in both treated and untreated groups compared to the control group. The findings of the present study offer compelling evidence for the association of inflammatory pathways with MDD, which is in agreement with previous studies [25, 26].

Untreated patients exhibited approximately five‐fold higher MYD88 gene expression compared to controls, whereas patients in the treated (remitted) group showed approximately three‐fold higher expression relative to controls. These cross‐sectional differences underscore the potential involvement of this adaptor protein in the TLR4/MyD88/NF‐κB signaling pathway in MDD. Recent studies have shown that activated microglia in the brains of depressed individuals release pro‐inflammatory cytokines through this pathway [27]. MYD88 acts as a key adaptor protein in Toll‐like receptor signaling, and its activation leads to the translocation of NF‐κB to the nucleus, resulting in the transcription of inflammatory genes such as TNF‐α, IL‐6, and IL‐1β—providing a mechanistic framework that may explain the higher MYD88 expression observed in untreated patients [28]. Although MYD88 expression was lower in the remitted group than in the untreated group, the cross‐sectional design of the study does not allow causal inferences regarding treatment‐related changes. Nevertheless, previous experimental and clinical studies suggest that antidepressants may exert anti‐inflammatory effects through modulation of the TLR4/MyD88/NF‐κB pathway [29].

Similarly, NF‐κB expression was higher in both patient groups compared with controls, with a greater elevation observed in untreated patients, highlighting the relevance of this transcription factor in inflammation‐related depression and aligning with reports of higher NF‐κB activity in MDD [30]. The observed between‐group differences should be interpreted as associative rather than indicative of treatment‐induced normalization. The absence of significant changes in TRIF gene expression, in contrast to MYD88, indicates the specificity of the inflammatory response in depression. Studies suggest that the MyD88‐dependent pathway plays a more dominant role in stress‐induced neuroinflammation [31].

The observed changes in serum levels of TNF‐α and IL‐6 are also consistent with previous research [32]. Untreated patients exhibited significantly higher TNF‐α levels than both controls and remitted patients, supporting the involvement of this cytokine in the inflammatory profile of active MDD. TNF‐α has been implicated in alterations of serotonergic signaling, hippocampal neurogenesis, and hypothalamic–pituitary–adrenal (HPA) axis regulation [33]. Recent studies have shown that pro‐inflammatory cytokines such as IL‐1α, IL‐6, and TNF‐α are associated with MDD, psychological distress, cardiovascular health, and obesity, which is consistent with our findings [33, 34]. Lower TNF‐α levels in the treated group should be interpreted as a characteristic of the remitted cohort rather than evidence of longitudinal reduction, although prior studies suggest that antidepressants may influence cytokine production through NF‐κB modulation [35].

The similar pattern observed for IL‐6, with a significant increase in the untreated group and a notable decrease in the treated group, aligns with the dual role of this cytokine in depression. Studies have shown that peripheral levels of IL‐6, IL‐10, IL‐12, IL‐13, and TNF‐α are higher in depressed patients compared to healthy controls [36]. In addition to its direct pro‐inflammatory effects, IL‐6 activates indoleamine 2,3‐dioxygenase (IDO), which leads to reduced tryptophan and, consequently, lower serotonin levels—a key mechanism in the pathogenesis of depression [37]. While IL‐6 levels differed between groups in the present study, causal relationships between treatment, cytokine modulation, and symptom improvement cannot be inferred from the cross‐sectional design. Nevertheless, experimental data indicate that inhibition of the TLR4/NF‐κB pathway may reduce IDO activity and alleviate depressive‐like behaviors [38, 39].

Based on previous literature, several hypotheses have been proposed to explain how inflammatory abnormalities contribute to the pathogenesis of major depression: (A) The Cytokine‐Induced Neurotransmitter Dysregulation Hypothesis; Pro‐inflammatory cytokines such as IL‐6 and TNF‐α reduce serotonin availability by activating the IDO pathway, decreasing tryptophan levels, and increasing neurotoxic kynurenine metabolites. This mechanism is well‐documented in meta‐analyses and clinical studies [5, 37]. (B) The Microglial Activation and Neuroinflammation Hypothesis; Chronic peripheral inflammation can activate microglia, leading to higher production of IL‐1β, TNF‐α, and reactive oxygen species in the CNS. This process disrupts synaptic plasticity, neurogenesis, and glutamate signaling, all strongly implicated in depression [40, 41]. (C) The HPA Axis Dysregulation Hypothesis; Cytokines stimulate the HPA axis, increasing cortisol release and impairing glucocorticoid receptor sensitivity. This leads to sustained stress‐response activation and contributes to depressive symptomatology [42]. Together, these hypotheses support the interpretation that inflammatory dysregulation is closely associated with MDD and may contribute to its maintenance or severity, although longitudinal and mechanistic studies are required to establish causality [43]. The present findings are consistent with this framework and highlight the relevance of inflammatory pathways in MDD.

This study also has limitations, including the lack of evaluation of other anti‐inflammatory cytokines such as IL‐10, the absence of an assessment of treatment duration on inflammation severity, and failure to control confounding factors such as BMI and nutritional status. Additionally, because all antidepressant‐treated patients were clinical remitters, comparisons between remitter and non‐remitter subgroups were not possible, limiting our ability to assess whether inflammatory markers differ according to treatment response status. As a result, the findings primarily reflect immunological characteristics of patients who achieved remission following antidepressant treatment and may not be generalizable to non‐responders or patients receiving alternative therapeutic regimens.

Furthermore, although group‐level comparisons of BDI scores and cytokine concentrations were performed, the study design did not allow for a detailed evaluation of individual‐level associations between clinical improvement and changes in inflammatory markers. This limitation restricts our capacity to determine whether reductions in depressive symptoms parallel decreases in pro‐inflammatory cytokines within subjects. Moreover, because the treated cohort consisted of patients identified during routine clinical follow‐up rather than through a prospective response‐stratified design, potential biological differences between responders and non‐responders could not be systematically examined.

Future studies should include both remitter and non‐remitter patients and incorporate longitudinal follow‐up during interepisode remission periods to better clarify the temporal dynamics of inflammatory changes in MDD. Furthermore, future studies should explore the association between different isoforms in inflammatory genes and treatment response, determine optimal cut‐off points for inflammatory biomarkers to predict treatment outcomes, and assess the efficacy of combining anti‐inflammatory agents with antidepressants.

## 5 Conclusion

The present cross‐sectional study indicates that patients with major depressive disorder exhibit significant alterations in selected inflammatory genes and circulating pro‐inflammatory cytokines compared with healthy individuals, supporting an association between immune dysregulation and MDD.

Given the cross‐sectional nature of the study, causal inferences regarding the effects of antidepressant treatment on inflammatory pathways cannot be drawn. Nevertheless, the observed group‐level differences suggest that inflammatory mechanisms may be differentially associated with disease status and treatment status. The findings of the present study highlight the utility of inflammatory biomarkers as adjunctive tools for patient stratification and clinical characterization. This may pave the way for the development of personalized therapeutic approaches based on patients' inflammatory profiles.

Accordingly, future studies should take the limitations of the present study into account and include larger and more diverse patient cohorts, examine longitudinal changes in inflammatory markers during interepisode remission, explore the relationship between clinical improvement and cytokine dynamics, and assess the potential benefits of combining anti‐inflammatory agents with standard antidepressant therapies.

## Author Contributions

**Yaser Mohammadi:** validation, visualization, writing – review and editing, formal analysis, software, writing – original draft. **Omid Kooshkaki:** conceptualization, methodology, data curation, writing – review and editing, validation. **Aliakbar Esmaeili:** conceptualization, investigation, visualization, methodology, writing – review and editing, data curation. **Hasti Anani Sarab:** investigation, data curation, software, writing – review and editing, visualization. **Gholamreza Anani Sarab:** conceptualization, investigation, funding acquisition, writing – original draft, methodology, validation, writing – review and editing, software, project administration, data curation, supervision.

## Ethics Statement

This study was conducted in accordance with the ethical standards of the Declaration of Helsinki. Ethical approval was obtained from the Ethics Committee of Birjand University of Medical Sciences (IR.BUMS.REC.1395.4664). Written informed consent was obtained from all participants prior to their enrollment in the study.

## Consent

The authors have nothing to report.

## Data Availability

The present study data are available from the corresponding author upon reasonable request.
